# Biosacetalin (1,1-Diethoxyethane) Improves Healthy Lifespan in *C. elegans* and Rats

**DOI:** 10.3390/antiox15020160

**Published:** 2026-01-24

**Authors:** Vu Hoang Trinh, Geun-Haeng Lee, Eun-Jong Kim, Jooyeon Sohn, Jin-Myung Choi, Thang Nguyen Huu, Dhiraj Kumar Sah, Sang-Chul Park, Min-Keun Song, Seung-Rock Lee

**Affiliations:** 1 Department of Biochemistry, Department of Biomedical Sciences, Chonnam National University Medical School, Gwangju 501190, Republic of Korea; 206847@jnu.ac.kr (T.N.H.); 197784@chonnam.edu (D.K.S.); 2 Department of Oncology, Department of Medical Sciences, Pham Ngoc Thach University of Medicine, Ho Chi Minh City 700000, Vietnam; 3 Alfred E. Mann School of Pharmacy and Pharmaceutical Sciences, University of Southern California, Los Angeles, CA 90033, USA; geunhaenglee@gmail.com; 4 Luxanima Inc., Room 102, 12-55, Sandan-gil, Hwasun-eup, Hwasun-gun 58128, Republic of Korea; choijm1@naver.com; 5 Department of Physical & Rehabilitation Medicine, Chonnam National University Medical School & Hospital, Gwangju 61469, Republic of Korea; trueisone@hanmail.net (E.-J.K.); drsongmk@jnu.ac.kr (M.-K.S.); 6 Department of Biological Sciences, Korea Advanced Institute of Science and Technology, 291 Daehak-ro, Yuseong-gu, Daejeon 34141, Republic of Korea; jooyeonsohn@kaist.ac.kr; 7 The Future Life & Society Research Center, Advanced Institute of Aging Science, Chonnam National University, Gwangju 61469, Republic of Korea; scpark@snu.ac.kr

**Keywords:** 1,1-diethoxyethane, NADH:ubiquinone oxidoreductase, ROS, signal pathways, anti-aging, longevity, memory

## Abstract

Recent evidence has highlighted the pivotal roles of reactive oxygen species (ROS) and the SIRT1, AMPK, and mTOR signaling pathways in aging and longevity, making them attractive targets for studies of lifespan-extending interventions. We previously demonstrated that 1,1-diethoxyethane (1,1-DEE) could interact with mitochondrial complex I (NADH–ubiquinone oxidoreductase), leading to transient mitochondrial ROS (mtROS) production and activation of the AMPK pathway. This study further examined the effects of 1,1-DEE on longevity in model organisms. Treatment with 1,1-DEE decreased senescence in endothelial cell EA.hy926. In *Caenorhabditis elegans* (*C. elegans*), 1,1-DEE induced a hormetic response and extended the lifespan, whereas its structural isoform, 1,2-diethoxyethane (1,2-DEE), showed no such effect. In rat models, administration of 1,1-DEE markedly improved survival rate, mortality risk, restricted mean survival time (RMST), and median lifespan, associated with an accelerated body weight reduction. Additionally, 1,1-DEE could also enhance learning and memory, as assessed by the Morris water maze test in rats. These findings suggest that 1,1-DEE may serve as a novel small-molecule modulator of mitochondrial function and redox signaling, with potentials for promoting anti-aging and longevity.

## 1. Introduction

Aging is a complex biological process characterized by a gradual decline in cellular and organ functions, which leads to increased susceptibility to age-related diseases such as cardiovascular diseases (CVDs), diabetes, osteoporosis, osteoarthritis, chronic obstructive pulmonary disease, and neurodegenerative disorders, including Alzheimer’s and Parkinson’s disease. This progressive functional deterioration not only affects individual health and quality of life but also imposes a substantial socioeconomic burden on modern societies. These challenges manifest as rising healthcare costs, shrinking working-age populations, and destabilization of pension systems. To address these issues, there has been a growing focus on understanding the molecular mechanisms underlying aging and developing interventions that can delay the aging process and promote healthy longevity in humans [1,2].

In longevity research, *Caenorhabditis elegans* has proven to be an invaluable model organism due to its relatively short lifespan of 2–3 weeks, allowing for the observation of the aging process over a compressed time frame. The molecular mechanisms governing aging in *C. elegans* share significant similarities with those in humans, particularly in terms of the insulin/IGF-1 signaling pathway, mitochondrial function, and protein homeostasis. Accordingly, *C. elegans* is an ideal model organism for investigating the molecular mechanisms of aging. Furthermore, *C. elegans* is cost-effective and can be easily cultured in large quantities, making it an efficient model for high-throughput research. Its simplicity also facilitates the testing of chemicals for potential toxicity or therapeutic effects, as the organism can be easily fed and treated with various substances. For these reasons, *C. elegans* continues to serve as a prominent model in longevity studies and drug development [3]. Interestingly, accumulating evidence has indicated that a modest inhibition of mitochondrial respiration can extend the lifespan of several species, including *C. elegans* [4]. In particular, mitochondrial complex inhibitors, such as metformin, and phloretin, have been demonstrated to prolong lifespan by inducing the production of mtROS, which in turn modulate aging-related signaling pathways [5,6].

Over the years, studies have identified key hallmarks of aging, which include impaired macroautophagy, chronic inflammation, dysbiosis, mitochondrial dysfunction, deregulation of nutrient sensing, loss of proteostasis, genomic instability, telomere attrition, epigenetic alterations, cellular senescence, stem cell exhaustion, and disruption in intercellular communication [7]. Recently, considerable progress has been made in elucidating the signaling pathways that influence aging and longevity [8]. Cellular signalings constitute a complex and interconnected network, allowing cells to coordinate responses to both intrinsic and extrinsic stimuli [9]. Among these signaling pathways, SIRT1, AMPK, and mTOR have garnered significant attention due to their individual roles, as well as their crosstalk, in regulating several aging hallmarks, such as autophagy, inflammation, mitochondrial function, and nutrient sensing. Notably, dysregulation of insulin/IGF-1, mTOR or AMPK signaling is strongly associated with aging and onset of age-related diseases. Interventions that modulate these pathways, such as physical exercise, caloric restriction, and administration of pharmacological agents (e.g., rapamycin and metformin), have demonstrated anti-aging effects and protection against age-related diseases [8,10].

Recently, we have identified 1,1-diethoxyethane (1,1-DEE) as a compound capable of interacting with mitochondrial complex I (NADH–ubiquinone oxidoreductase) and inducing transient generation of mtROS. Treatment with 1,1-DEE was shown to increase insulin sensitivity and ameliorate obesity and dyslipidemia in the high-fat diet-fed animal model, and the parallel study demonstrated that it also activates the AMPK signaling pathway [11,12,13]. Based on these findings, the present study further examined the potential of 1,1-DEE in longevity.

## 2. Materials and Methods

### 2.1. Cell Experiments

#### 2.1.1. Cell Culture and Treatment

EA.hy926 human endothelial cells were obtained from the American Type Culture Collection (CRL-2922; ATCC, Manassas, VA, USA) and cultured in Dulbecco’s Modified Eagle Medium (DMEM) (LM001-05; Welgene, Gyeongsan, Republic of Korea) supplemented with 10% heat-inactivated fetal bovine serum (S001-01; Welgene, Gyeongsan, Republic of Korea) and 1% Penicillin–Streptomycin (LS 202-02; Welgene, Gyeongsan, Republic of Korea). Cells were maintained at 37 °C in a humidified incubator with 5% CO_2_. Cells were starved overnight with serum-free DMEM before experiment. For treatment, cells were preconditioning with various concentrations of 1,1-DEE for 8 h and subsequently incubated with the presence of 100 μg/mL oxidized-low density lipoprotein (ox-LDL) (L34357; ThermoFisher, Waltham, MA, USA) for 48 h. This ox-LDL regimen was selected based on previously described protocol for assessing cellular senescence in EA.hy926 cells [14].

#### 2.1.2. Senescence-Associated β-Galactosidase Staining

Cellular senescence was assessed using a Senescence β-Galactosidase (SA-β-gal) staining kit (9860; Cell Signaling Technology, Danvers, MA, USA), according to the manufacturer’s instructions. Briefly, culture media were removed, and cells were washed once with 1× phosphate-buffered saline (PBS) then fixed in 1× fixative solution for 15 min at room temperature. After fixation, cells were rinsed with PBS and incubated with the β-galactosidase staining solution for 18–24 h at 37 °C. Subsequently, cells were examined under a light microscope, and senescent cells were identified as those exhibiting blue cytoplasmic staining. The percentages of β-galactosidase positive cells were calculated. Data are expressed as mean ± SEM of at least three independent experiments.

### 2.2. C. elegans Model

#### 2.2.1. Development of *C. elegans*

In this study, the wild-type *C. elegans* strain, Bristol N2, was used as previously reported [15,16]. Bleached eggs were cultured in liquid medium to the L1 larval stage at 20 °C by adding 1,1-DEE or 1,2-DEE to the S-basal buffer. During culture, the bleached eggs were placed in 50 mL tubes and mounted on a rotator, where they were gently rotated at a slow speed to ensure uniform mixing. Once the worms reached the L1 larval stage, they were separated from the S-basal buffer by centrifugation and transferred to liquid nematode growth medium (NGM). *Escherichia coli* OP50, the food source for the worms, was added to the liquid NGM. The worms were then distributed into a 96-well plate. Each well contained a total volume of 200 µL, consisting of liquid NGM, OP50, approximately 20 worms, 1,1-DEE, or 1,2-DEE (0, 100 µM, 1 mM, 20 mM), and amphotericin B (0.1 µg/mL), which prevents fungal contamination. The 96-well plate was placed on a shaker to gently mix the contents and prevent drug sedimentation. The plate was then cultured at 20 °C. After 2–3 days of incubation, the developmental state of the worms was observed using a microscope, and the effects of 1,1-DEE or 1,2-DEE were assessed.

#### 2.2.2. Longevity of *C. elegans*

The setup for the lifespan experiment was identical to that for the development experiment, with the addition of 5-fluoro-2′-deoxyuridine (FUDR), which prevents progeny hatching, when *C. elegans* larvae reached the L4 stage. Each well contained a total volume of 200 µL, consisting of liquid NGM, OP50, approximately 20 worms, 1,1-DEE or 1,2-DEE (0, 100 µM, 1 mM, 20 mM), amphotericin B (0.1 µg/mL), and FUDR (200 µM). The 96-well plate was placed on a shaker to gently mix the contents and prevent the settling of the drugs, and it was cultured at 20 °C. Worm survival was monitored every 2–3 days, and the worms were considered dead when no movement was observed in the liquid medium. All experiments were independently performed by two different experimenters.

### 2.3. Rat Model

#### 2.3.1. Survival of Rats

Sprague-Dawley (SD) male rats (12-month-old, *n* = 22) were obtained from Samtako (Osan, Republic of Korea). The animals were housed under controlled conditions with a 12 h light/dark cycle and had ad libitum access to standard chow and water. Prior to the experiment, the animals were acclimated for 2 weeks. The rats were randomly assigned to either control or treatment group. At the beginning of the experiment, all animals received a single intravenous injection via the tail vein. The control group was administered normal saline, whereas the treatment group received 1,1-DEE at a dose of 300 mg/kg. Two rats died within hours following drug injection, before the start of the survival observation period. These animals were therefore excluded from the experiment, and final analyzed cohort consisted of 10 rats per group. The rats were monitored for 48 weeks in uniform feeding and living conditions, and body weights were recorded weekly. All experimental procedures were performed in accordance with the Guidelines for the Care and Use of Laboratory Animals of Chonnam National University (CNUH IACUC-22020 and CNU IACUC-H-2025-87).

#### 2.3.2. Spatial Learning and Memory of Rats

Morris water maze, a popular and reliable test for assessing spatial learning and memory in rodents [17], was employed to obtain Supplementary Data in this study. The experiment was performed using the same strain of rats as described above. A circular pool (diameter: 180 cm, height: 60 cm) was placed in a laboratory with controlled lighting and curtains to prevent external distractions and filled to a depth of 40 cm with room temperature water (25 ± 1 °C), made opaque by adding non-toxic gray tempera paint. The pool was conceptually divided into four quadrants clockwise (Zone 1, 2, 3, and 4), with an escape platform in Zone 3. During each trial, rats were released into the pool from Zone 1 and given up to 60 s to locate the platform. Rats that failed to reach the platform within the allotted time were gently guided to it and allowed to remain there for 10 s to orient themselves. Rats that successfully located the platform were also allowed to remain for 10 s. After each trial, animals were dried with a soft towel and placed in a pre-warmed holding cage. Testing was performed at a consistent time of day to minimize circadian variation. In preparation for the experiment, twelve old SD rats (22-month-old) underwent one day of training for adapting to the environment and learning the task. To start, rats were randomly assigned into two groups: control (*n* = 6) and treatment (*n* = 6); and one day before the first test, each group received an intravenous tail vein injection of either normal saline or 1,1-DEE (20 mg/kg). Three testing sessions were conducted: the first test was performed one day after injection (day 1), the second test four days later (day 5), and the third test four days after the second session (day 9). The number of rats succeeded in reaching platform within 60 s in each group was manually recorded to assess memory retention across the testing sessions.

### 2.4. Chemical Properties

Data on the chemical properties of the compounds were obtained from the ChemSpider Search and share chemistry of Royal Society of Chemistry (https://www.chemspider.com).

### 2.5. Statistical Analysis

Quantitative variables are presented as the mean ± SEM. For comparisons between two groups with normal distribution, unpaired *t* tests were applied. Log-rank test, Cox regression and portable version of Online application for survival analysis (OASIS) [18] were used for survival analysis. Percentage changes in lifespan were compared via the area under curve (AUC) and the restricted mean survival time (RMST) [19]. Statistical comparisons were performed using R software (version 4.5.0; R Foundation for Statistical Computing, Vienna, Austria). *p* value less than 0.05 indicates a statistically significant difference.

## 3. Results

### 3.1. Chemical Properties of 1,1-DEE and Isoform 1,2-DEE

We analyzed the chemical characteristics of 1,1-DEE, its isoform 1,2-DEE, as well as metformin and resveratrol. In addition to molecular formulas and molecular weights, we compared two critical parameters relevant to drug absorption: logP and polar surface area (PSA). LogP, defined as the base-10 logarithm of a compound’s partition coefficient between octanol and water, serves as a key indicator of membrane permeability. Compounds with negative logP values are highly hydrophilic and typically exhibit poor passive membrane diffusion, whereas higher logP values indicate lipophilicity and greater absorption potential [20]. The PSA also offers predictive insights into the ability of small molecules to cross biological membranes, with lower PSA values generally associated with better passive diffusion [21]. These properties provide valuable information for assessing the membrane permeability and absorption potential of 1,1-DEE and its isoform in comparison with established agents such as metformin and resveratrol (Table 1).

### 3.2. Anti-Senescent Effect of 1,1-DEE in EA.hy926 Endothelial Cells

**Figure 1:**
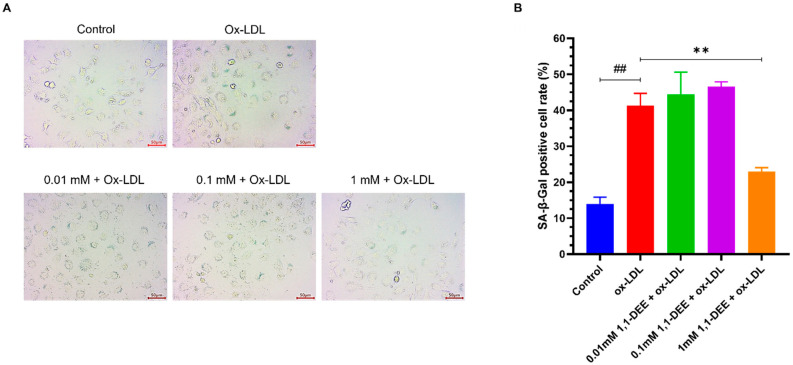
Protective effect of 1,1-DEE against ox-LDL–induced senescence in EA.hy926 endothelial cells. EA.hy926 human endothelial cells were preconditioned with 1,1-DEE (0, 0.01, 0.1, or 1 mM) for 8 h and subsequently exposed to ox-LDL for 48 h. Cellular senescence was assessed by SA-β-gal staining. (**A**): Cell images were observed via light microscope at 200×. (**B**): Significant reduction in senescent rate following 1 mM 1,1-DEE preconditioning (*t*-test, ^##^ *p*<0.01, ** *p* < 0.01). Data are presented as mean ± SEM from at least three independent experiments.

To investigate whether 1,1-DEE exerts protective effect against the senescence and age-related diseases, EA.hy926 human vascular endothelial cells were exposed to oxidized LDL. Oxidized LDL is known to induce endothelial senescence by generating excessive ROS and activating inflammatory pathways, consequently promoting atherosclerosis, an age-related disease that remains the leading cause of mortality [22]. In the experiment, cells were pre-conditioned with various concentrations of 1,1-DEE (0, 0.01, 0.1, 1 mM) for 8 h, followed by incubation with 100 µg/mL ox-LDL for 48 h. Senescence was assessed using SA-β-gal staining. Without treatment, ox-LDL markedly increased the proportion of senescent cells by approximately three-fold compared with the control. (41.3% vs. 14%, *t* test, *p* < 0.01). In contrast, preconditioning with 1 mM 1,1-DEE significantly suppressed ox-LDL–induced senescence, reducing the rate by approximately 44.3% (from 41.3% to 23.0%, *t* test, *p* < 0.01). The standardized effect size was very large (Cohen’s d = 4.1), indicating a strong biological impact of 1,1-DEE treatment. Lower concentrations of 1,1-DEE did not exhibit any noticeable difference (Figure 1).

### 3.3. Toleration of 1,1-DEE in C. elegans at Specific Concentrations

**Figure 2:**
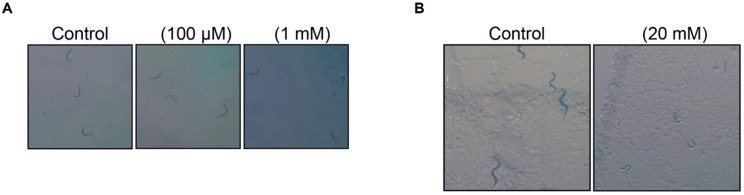
Effect of 1,1-DEE on the development of *C. elegans*. (**A**): Normal development of *C. elegans* treated with 1,1-DEE (100 μM and 1 mM). (**B**): Developmental inhibition following exposure to a higher concentration of 1,1-DEE (20 mM).

Before assessing its impact on longevity, we investigated whether 1,1-DEE had any adverse effects on the development of *C. elegans*. Exposure of *C. elegans* to 100 μM or 1 mM 1,1-DEE did not result in any noticeable developmental abnormalities. However, a higher concentration of 1,1-DEE (20 mM) inhibited the development of *C. elegans* (Figure 2). Based on these results, we selected 100 μM and 1 mM for the lifespan experiment, which were considered safe for the development of *C. elegans*.

### 3.4. Effect of 1,1-DEE on the Lifespan of C. elegans

**Figure 3:**
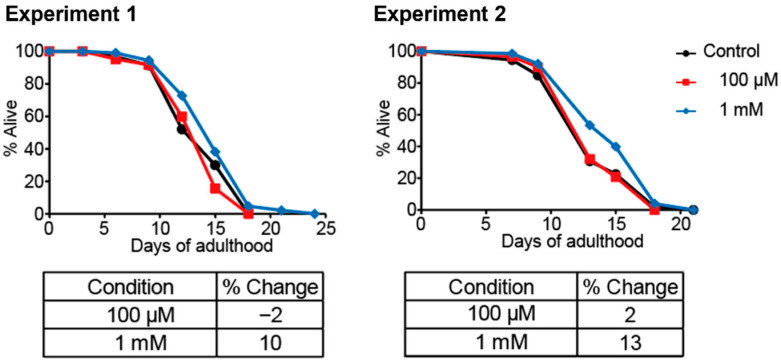
Lifespan of *C. elegans* following treatment with 1,1-DEE. Treatment at 100 μM had minimal effects, whereas 1 mM significantly extended lifespan (*p* < 0.05). Data represent two independent experiments.

The lifespan of *C. elegans* was assessed using a liquid-based cultivation system at two concentrations of 1,1-DEE (100 μM and 1 mM). The experiments were independently conducted by two experimenters. Treatment with 100 μM 1,1-DEE resulted in minimal changes in lifespan (−2% and 2%), which were not statistically significant. In contrast, 1 mM 1,1-DEE significantly extended the lifespan of *C. elegans* by approximately 10% in experiment 1 and 13% in experiment 2 (*p* < 0.05) (Figure 3).

### 3.5. Effect of Isoform 1,2-DEE on the Lifespan of C. elegans

**Figure 4:**
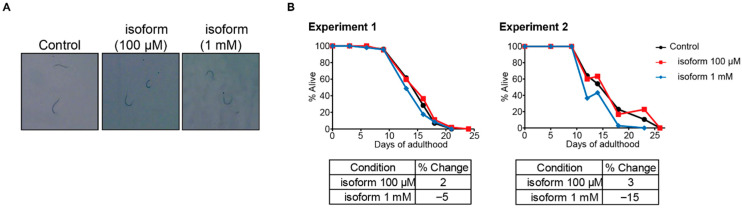
Effect of isoform 1,2-DEE on *C. elegans*. (**A**): Normal development of *C. elegans* treated with the isoform (100 μM and 1 mM). (**B**): No significant lifespan extension at either concentration.

We also investigated the effect of isoform 1,2-DEE on the lifespan of *C. elegans*, using the same concentrations as those in lifespan assays with 1,1-DEE (100 μM and 1 mM). Neither concentration of the isoform induced developmental abnormalities. However, neither concentration significantly extended the lifespan of *C. elegans*. These results indicated that pro-longevity effects may be specific to 1,1-DEE (Figure 4).

### 3.6. Effect of 1,1-DEE on Survival in SD Rats

**Figure 5:**
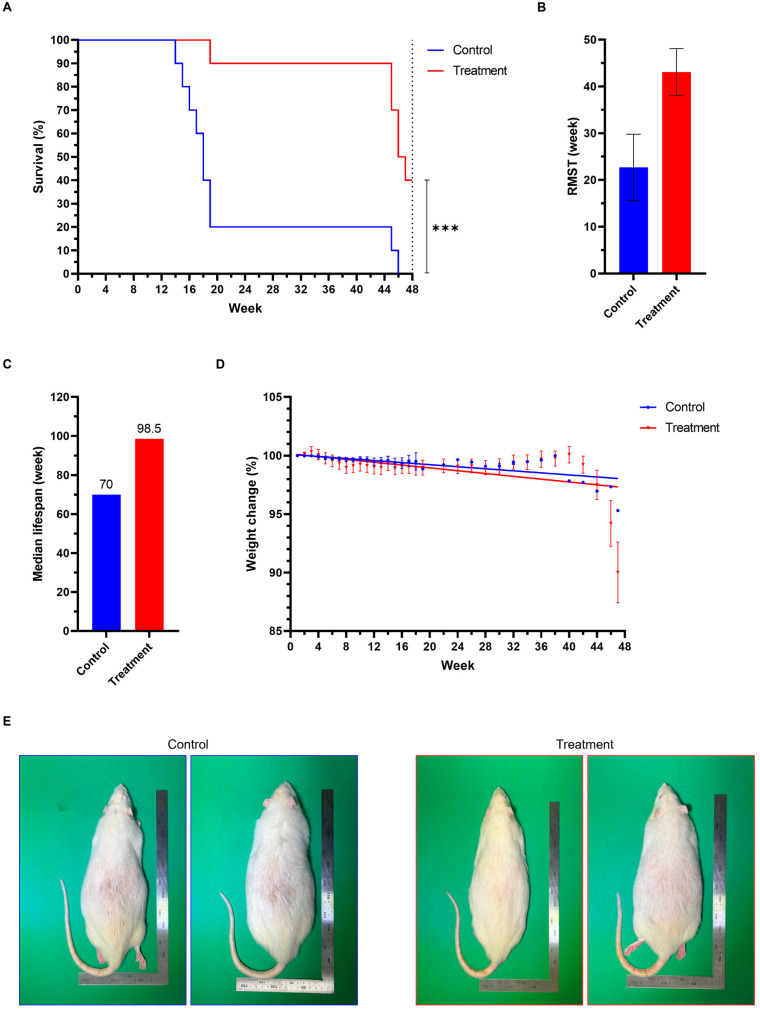
Effect of 1,1-DEE on survival and body weight in SD rats within 48-week observation period. (**A**): Survival analysis showing a significantly higher survival rate in the 1,1-DEE group compared with the control group (40% vs. 0%; log-rank test, *** *p* < 0.001). Cox proportional hazards regression showed an 85% reduction in mortality risk (HR = 0.15, 95% CI: 0.05–0.46). (**B**) The RMST of the treatment group was 43.1 weeks (95% CI: 38.1–48.1), compared with 22.7 weeks (95% CI: 15.6–29.8) in the control group, corresponding to a 20.4-week gain (1.9-fold extension). (**C**): The treatment group showed a longer median lifespan of 98.5 weeks compared to 70 weeks in controls. (**D**): Linear regression analysis showing a faster decline in body weight in the treatment group. (**E**): Representative images of our rat model.

We also assessed the effect of 1,1-DEE on lifespan in a rat model. Over the 48-week observation period, the survival rate in the treatment group was significantly higher than that in the control group (40% vs. 0%; log-rank test, *** *p* < 0.001). Cox proportional hazards analysis demonstrated that administration of 1,1-DEE reduced mortality risk by approximately 85% (HR = 0.15, 95% CI: 0.05–0.46) (Figure 5A). To compare survival time, we calculated the RMST [19]. RMST of the treatment group was 43.1 weeks (95% CI: 38.1–48.1), which was 20.4 weeks longer than that of the control group (22.7 weeks; 95% CI: 15.6–29.8) (Figure 5B). This corresponds to a 1.9-fold extension in survival time. Moreover, animals treated with 1,1-DEE exhibited a higher median lifespan (98.5 weeks) compared with controls (70 weeks), suggesting a potential benefit in longevity (Figure 5C).

### 3.7. Effect of 1,1-DEE on Weight Reduction in SD Rats

We performed linear regression analysis to assess weight changes over time in both groups. In the control group, the regression equation was y = −0.044x + 100.1, with a slope significantly different from zero (−0.044, *p* < 0.0001; 95% CI: −0.055 to −0.032). In the treatment group, the regression equation was y = −0.060x + 100.2, with a steeper slope that was also significantly different from zero (−0.060, *p* < 0.0001; 95% CI: −0.081 to −0.039), indicating a faster decline in body weight. These results suggest that 1,1-DEE treatment may accelerate body weight reduction (Figure 5D).

## 4. Discussion

For decades, reactive oxygen species (ROS) were primarily viewed as a driving force of aging and age-associated pathologies, a concept known as “Free Radical Theory of Aging” or “Mitochondrial Theory of Aging”. However, recent research has shifted this paradigm, highlighting the beneficial role of physiological ROS in promoting longevity. Evidence from model organisms has demonstrated that ROS can function as key signaling molecules that contribute to lifespan extension [23]. Mitochondrial complex I, a crucial regulator of cellular energy and redox balance, plays an important role in this process. Inhibition of complex I leads to reduced ATP levels and an increased AMP/ATP ratio, thereby activating AMPK and subsequently promoting mitochondrial biogenesis [24]. Several lifespan-extending compounds act by inhibiting complex I, triggering the generation of mtROS and initiating mitohormesis—an adaptive stress response that enhances cellular defense mechanisms. Such ROS-mediated signaling has also been implicated in the beneficial effects of calorie restriction, hypoxia, thermal stress, and exercise, as well as in pathways downstream of insulin/IGF-1 receptors, AMPK, TOR, and sirtuins, all of which contribute to proteostasis, unfolded protein response, stem cell maintenance, and stress resistance. Notably, mtROS-mediated signaling can extend lifespan even independently of AMPK [5]. Our previous studies have provided mechanistic evidence that 1,1-diethoxyethane (1,1-DEE) interacts with mitochondrial complex I, induces transient mtROS production, and activates AMPK. These mechanistic effects underlie its capability to enhance insulin sensitivity and alleviate obesity and dyslipidemia in an animal model [11]. Given that the mechanistic pathways of 1,1-DEE resemble those of metformin and resveratrol, two well-established compounds in longevity research, we sought to evaluate its potential for lifespan extension.

According to the World Health Organization, CVDs are the leading cause of mortality and premature death [25], for which obesity and dyslipidemia are major risk factors. Elevated circulating LDL, particularly oxidized LDL, accelerates endothelial senescence, thereby hastening the progression of atherosclerosis [22]. Consequently, atherosclerosis increases the incidence of cardiovascular diseases, including myocardial infarction, stroke, and heart failure, ultimately contributing to premature mortality and reduced lifespan. Following our previous evidence supporting the beneficial effects of 1,1-DEE on obesity and dyslipidemia, we examined its impact on senescence in endothelial cells. The result demonstrates that specific concentration of 1,1-DEE effectively attenuated LDL-induced endothelial senescence, suggesting a potential protective role against age-related CVDs and mortality (Figure 1).

For the longevity study, we investigated the effects of 1,1-DEE in *C. elegans* model. In the dose–response assessment, a high concentration (20 mM) impaired development, while 1 mM extended lifespan, and 100 μM showed no impairment (Figure 2). These results suggest a pattern typical of pharmacological and lifestyle interventions that promote longevity. This biphasic dose–response relationship, characterized by low-dose stimulation and high-dose inhibition, is a hallmark of hormesis, commonly observed with longevity-promoting compounds. The hormetic response may arise from direct stimulation or mild stress that transiently disrupts homeostasis, triggering an adaptive overcompensation response that enhances cellular repair and defense [26,27]. For instance, resveratrol demonstrates a hormetic dose–response in multiple biological models, affecting endpoints of biomedical and therapeutic relevance [28]. In particular, it can extend lifespan at low concentrations and induce toxicity at high concentrations [29]. Similarly, metformin can induce hormesis with the generation of ROS, extending both the lifespan and healthspan of *C. elegans* [30]. Consistent with these findings, 1,1-DEE exhibited a hormetic response associated with ROS generation, positioning it as a “hormetin” with potential benefits for healthy lifespan extension [31]. As observed in our study, 1 mM 1,1-DEE extended the lifespan of *C. elegans* by 10% and 13% (Figure 3), a result consistent with a meta-analysis indicating that hormetic effects could increase the mean lifespan of wild-type *C. elegans* by approximately 16.7% [32].

The concept of mitochondrial hormesis or mitohormesis, introduced by Tapia, posits that sublethal mitochondrial stress, accompanied by a modest increase in ROS, can induce beneficial changes in cellular physiology. These benefits can be achieved through interventions such as caloric restriction, intermittent fasting, exercise, or dietary phytonutrients [33]. However, some longevity studies have shown that pro-oxidant compounds, which exogenously generate ROS, may affect lifespan but in an unpredictable manner. It is suggested that ROS generated from mitochondria play the role in lifespan determination [23]. mtROS may serve as signaling molecules that transmit responsive signals from the mitochondria to other cellular compartments, thereby eliciting adaptive responses [34]. These mitohormetic ROS signals are usually temporary and may dissipate at steady-state levels [5]. The precise ROS responsible for promoting longevity remain unclear. Ludovico et al. suggested that H_2_O_2_ may play a key role in inducing hormesis [35]. However, the mechanisms by which a small and transient amount of physiological H_2_O_2_ can function as a signaling molecule, especially in a cellular environment rich in antioxidants, remain a subject of debate. Peroxymonocarbonate, a highly reactive but short-lived oxidant formed by the reaction of H_2_O_2_ with bicarbonate, has been suggested to play an important role in ROS signaling pathways [36]. In a parallel study, we found that 1,1-DEE-induced oxidation depends on the presence of bicarbonate, implying that the mitohormetic effects of 1,1-DEE may be mediated by the formation of peroxymonocarbonate.

To interact with mitochondrial complexes and induce mtROS production, it is apparent that compounds must pass through cellular membranes to reach the mitochondria. However, in case of metformin, it exhibits poor passive membrane permeability due to its hydrophilic nature (logP = −2.31) and high PSA (89 Å^2^), thereby relies on active uptake via organic cation transporters [37]. In contrast, 1,1-DEE is more lipophilic (logP = 1.15) and has a lower PSA (18 Å^2^), suggesting superior membrane permeability. Comparing with resveratrol, 1,1-DEE may achieve better membrane penetration owing to its smaller molecular size (118.176 Da vs. 228.247 Da) and lower PSA (18 Å^2^ vs. 61 Å^2^). Furthermore, a structural comparison between the two isomers highlights functional distinctions: 1,1-DEE is a geminal acetal (CH_3_–CH(OC_2_H_5_)_2_), whereas 1,2-DEE contains a dialkyl ether moiety (C_2_H_5_O–CH_2_–CH_2_–O–C_2_H_5_) (Table 1). In our study, only 1,1-DEE but not 1,2-DEE extended the lifespan of *C. elegans*, suggesting that lifespan-extending effects may be closely associated with acetal functionality rather than the ether moiety or diethoxy substitution (Figure 4).

In the context of promoting health and lifespan through mitohormesis, transiently elevated ROS levels may provide a “vaccination-like” mechanism that enhances adaptive responses and cellular defense [5]. Preconditioning, as a condition of hormesis, has been demonstrated in several studies [38]. Thus, in our rat model, we applied 1,1-DEE as a preconditioning agent by administering a single sublethal dose to induce a transient oxidative stress. Over 48-week period, this intervention resulted in a significantly higher survival rate in the treatment group than in the control group (40% vs. 0%; log-rank test, *** p < 0.001), accompanied by a 1.9-fold increase in RMST and an 85% reduction in mortality risk (HR = 0.15). Consistently, median lifespan was extended from 70 to 98.5 weeks. These findings demonstrate a pro-longevity effect in a mammalian model, paralleling the lifespan-extending effects observed in *C. elegans* (Figure 5). Notably, by 20 weeks observation, unexpected deaths were recorded, especially in control group. At this time point, all rats were approximately 17 months of age (late middle-aged), which is slightly below the typical median lifespan of SD rats [39]. Thus, this substantial mortality may represent premature deaths rather than natural lifespan limits. Some factors may have contributed to this outcome, including stress associated with transfer to a new feeding and housing conditions or drug injection. Moreover, at the beginning, the baseline body weight of the experimental cohort was markedly elevated. At around 50 weeks of age, mean body weight was 753.6 ± 14.7 g (mean ± SEM), significantly exceeding the reference value of 579.1 g for age-matched male SD rats [40] (753.6 g vs. 579.1 g, 30.1% higher, one sample *t*-test, *p*< 0.01). This reflects an overweight status, which is known to increase susceptibility to metabolic dysfunction and age-associated mortality. Interestingly, we found that 1,1-DEE accelerated body weight reduction (Figure 5D), consistent with prior findings showing that most longevity-enhancing compounds are associated with reduced body weight [41]. The weight reduction in the 1,1-DEE group was associated with a significant decrease in mortality risk (85%) and an extension of median life span (up to 98.5 weeks). Given that the control group suffered from the premature mortality likely due to high baseline body weight, we interpret the 1,1-DEE induced weight loss as a protective metabolic adjustment rather than a toxic effect. This effect mirrors caloric restriction, suggesting a link between the aging process and changes in muscle or adipose tissue [42]. Taken together, although the premature deaths lead to early separation of survival curves and limit definitive conclusions regarding maximum lifespan extension, the combined evidence from survival rate, RMST, hazard ratio and median lifespan support that 1,1-DEE confers a protective effect on survival and health span, particularly under conditions predisposing to premature mortality.

As memory decline is a common feature of the aging process, the effects of aging on memory and brain function have been extensively studied [43]. In contrast, dementia disorders also contribute to increased mortality and reduced lifespan [44]. Interventions capable of extending lifespan in animal models, including caloric restriction, physical exercise and pharmacological agents (such as resveratrol, rapamycin, and metformin), may positively influence brain cognition [45]. In this context, we conducted a preliminary experiment in old rats to investigate the potential of 1,1-DEE on spatial learning and memory. Morris water maze performance indicated that the treatment group maintained a 50% success rate across testing days, whereas the control group failed entirely (Figure S1). These results provide preliminary evidence that 1,1-DEE may enhance memory retention, warranting future in-depth examinations.

Although our study provides important insights, it has some limitations. We did not directly investigate all underlying cellular mechanisms, as we derived data from our parallel studies. In the rat model, the observation period was limited to 48 weeks, preventing comparisons of the maximum lifespan. Moreover, because formal necropsy was not performed, the precise causes of death could not be determined, and therefore the contribution of non–aging-related mortality cannot be excluded. Future studies employing expanded survival analyses and mechanistic investigations in longevity models will be necessary to further substantiate lifespan-extending effect of 1,1-DEE.

In conclusion, building upon our previous findings demonstrating that 1,1-DEE could interact with mitochondrial complex I (NADH–ubiquinone oxidoreductase), generating mtROS and subsequently activating key cellular signaling pathways, we propose that 1,1-DEE may exert mitohormetic effects that promote longevity. The results of the present study support that hypothesis: treatment with 1,1-DEE could decrease endothelial cell senescence, extend the lifespan of *C. elegans* and improve the survival rate, mortality risk, RMST and median lifespan of rats. Looking forward, given that established lifespan-extending compounds such as metformin and resveratrol also exhibit protective effects in neurodegenerative and cardiovascular diseases [46], 1,1-DEE may hold promise as an intervention for age-related degenerative disorders. We propose Biosacetalin as the common name of 1,1-diethoxyethane, meaning a bioactive acetal beneficial for human health and disease.

## Figures and Tables

**Table 1:** Chemical properties of 1,1-DEE, isoform 1,2-DEE, metformin, and resveratrol.

Name	Molecular Formula	Condensed Structural Formula	MW (g/mol)	LogP	PSA (Å^2^)
1,1-Diethoxyethane 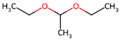	C_6_H_14_O_2_	CH_3_–CH(OC_2_H_5_)_2_	118.176	1.15	18
1,2-Diethoxyethane 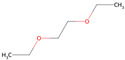	C_6_H_14_O_2_	C_2_H_5_O–CH_2_–CH_2_–OC_2_H_5_	118.176	0.62	18
Metformin	C_4_H_11_N_5_	H_3_C–N–C(=NH)–N–C(=NH)–N–CH_3_	129.167	−2.31	89
Resveratrol	C_14_H_12_O_3_	HO–C_6_H_3_–CH=CH–C_6_H_2_–OH_2_	228.247	3.14	61

## Data Availability

The data presented in this study are available on request from the corresponding authors.

## Supplementary Materials

The following supporting information can be downloaded at: https://www.mdpi.com/article/10.3390/antiox15020160/s1, Figure S1: Effect of 1,1-DEE on spatial cognition and memory.
