# Supplementary material for: Biosacetalin (1,1-Diethoxyethane) Improves Healthy Lifespan in C. elegans and Rats
Source: Antioxidants (Basel). 2026 Jan 24;15(2):160. doi: 10.3390/antiox15020160 (PMC12937443; doi:10.3390/antiox15020160)
Supplement: Supplementary file 1 [file antioxidants-15-00160-s001.zip › antioxidants-4025392-supplementary.pdf]

antioxidants

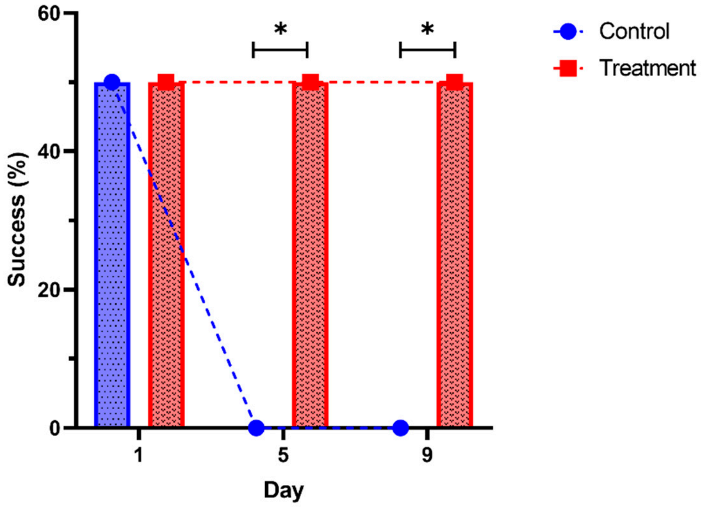

Figure S1. ***Effect of 1,1-DEE on spatial cognition and memory.*** *Performance of SD rats in Morris water maze test demonstrated enhanced memory retention in treatment group compared to controls. On day 1, both groups showed equivalent results, with 50% rats successfully locating the platform. However, by day 5 and day 9, the success rates of control group declined to 0%, whereas the 1,1-DEE–injected group maintained a consistent 50% success rate (Chi-square test, p = 0.046).*
